# Postural Control in Dual-Task Situations: Does Whole-Body Fatigue Matter?

**DOI:** 10.1371/journal.pone.0147392

**Published:** 2016-01-21

**Authors:** Rainer Beurskens, Matthias Haeger, Reinhold Kliegl, Kai Roecker, Urs Granacher

**Affiliations:** 1 Research Focus Cognition Sciences, Division of Training and Movement Sciences, University of Potsdam, Potsdam, Germany; 2 Department of Psychology, Division of Cognitive Psychology, University of Potsdam, Potsdam, Germany; 3 Faculty of Applied Public Health, University of Furtwangen, Furtwangen, Germany; 4 Geriatric Center at the University of Heidelberg, Agaplesion Bethanien Hospital Heidelberg, Heidelberg, Germany; Center for BrainHealth, University of Texas at Dallas, UNITED STATES

## Abstract

Postural control is important to cope with demands of everyday life. It has been shown that both attentional demand (i.e., cognitive processing) and fatigue affect postural control in young adults. However, their combined effect is still unresolved. Therefore, we investigated the effects of fatigue on single- (ST) and dual-task (DT) postural control. Twenty young subjects (age: 23.7 ± 2.7) performed an all-out incremental treadmill protocol. After each completed stage, one-legged-stance performance on a force platform under ST (i.e., one-legged-stance only) and DT conditions (i.e., one-legged-stance while subtracting serial 3s) was registered. On a second test day, subjects conducted the same balance tasks for the control condition (i.e., non-fatigued). Results showed that heart rate, lactate, and ventilation increased following fatigue (all *p* < 0.001; *d* = 4.2–21). Postural sway and sway velocity increased during DT compared to ST (all *p* < 0.001; *d* = 1.9–2.0) and fatigued compared to non-fatigued condition (all *p* < 0.001; *d* = 3.3–4.2). In addition, postural control deteriorated with each completed stage during the treadmill protocol (all *p* < 0.01; *d* = 1.9–3.3). The addition of an attention-demanding interference task did not further impede one-legged-stance performance. Although both additional attentional demand and physical fatigue affected postural control in healthy young adults, there was no evidence for an overadditive effect (i.e., fatigue-related performance decrements in postural control were similar under ST and DT conditions). Thus, attentional resources were sufficient to cope with the DT situations in the fatigue condition of this experiment.

## Introduction

Postural control is important to cope with the demands of our environment (e.g., walking on cobblestones) and to minimize the risk of falls [1]. Additionally, postural control is important in many sport disciplines (e.g., gymnastics). A large body of literature has illustrated that attentional demand is needed to control posture [2–4]. In young healthy adults, postural sway increased during one-legged stance while concurrently performing a verbal (e.g., spelling words) [2] or arithmetic (e.g., serial subtraction [4, 5]) interference task. These findings clearly indicate that attentional capacity is required to maintain postural control.

There is ample evidence that postural control is affected by fatigue. Many studies examined the influence of fatiguing lower extremity muscles [6–8], and they all showed impairments in measures of postural control [9]. Zech et al. [7] examined the effects of whole-body fatigue on postural sway in young adults and found deteriorated one-legged stance performance after completion of an incremental all-out treadmill test. In general, fatigue-related decrements in motor performance develop due to peripheral changes at the level of the muscle and because of insufficient drive of the central nervous system to the motor neurons [10]. Peripheral mechanisms include changes in the neuromuscular junction, the sarcolemma, accumulation of metabolites and depletion of substrates, which ultimately results in a reduced ability of muscles to apply forces. Central factors comprise impaired cortico-spinal transmission to the spinal cord, reduced motor neuron excitability and firing frequency [11]. The afferent side is affected by diminished synaptic feedback following fatigue (i.e., decreased H-reflex amplitude [11]). Lastly, proprioceptive (i.e., errors in perceived joint position and force generation) [12] and motivational aspects (i.e., loss of motivation and attentional focus) [13] impair motor performance. The mentioned mechanisms are well-suited to also explain decrements in postural control following whole-body fatigue. However, a direct relationship between those fatigue-related mechanisms and deteriorations in postural control has not been established yet. Whole-body exercises primarily demand energetic metabolism and are better suited to mimic everyday-like requirements than localized muscle fatigue, which strongly stimulate the neuromuscular system [9].

Fatigue also influences cognitive performance [14], and affects performance in dual-task (DT) situations [15–17]. Simoneau and colleagues examined the effects of a whole-body fatigue protocol on DT balance performance in young adults [15]. Participants simultaneously performed a dynamic balance control task and a reaction time task before and after three periods of moderate fatigue (fast walking on a treadmill). Fatigue resulted in impaired balance performance and increased attentional demand suggesting that more cognitive resources had to be allocated to the balance task during the fatigued compared to the non-fatigued condition. This increase in cognitive demand is well-suited to interfere with cognitive processing during DT motor performance. Also, it is still unresolved how gradually increasing levels of fatigue (incremental treadmill protocol) affect single-task (ST) and DT balance performance and how exaggerated breathing movements, which typically occur during whole-body fatigue [18, 19], influence postural control.

Thus, the aim of the present study was to examine the impact of whole-body fatigue on ST and DT postural control in young healthy adults. We hypothesize that a) postural control deteriorates with gradually increasing levels of fatigue, and b) these impairments are more pronounced during DT situations.

## Methods

### Ethics statement

The Human Ethics Committee at the University of Potsdam approved the study protocol (approval number: 49/2014). Before the start of the study, each participant read, concurred, and signed a written informed consent. All procedures were conducted according to the Declaration of Helsinki.

### Participants

Twenty healthy adults participated in our experiments (Table 1). None of them had any known neuromuscular or orthopaedic diseases/injuries that may have affected their ability to conduct the experiments. In addition, all participants were naïve with regards to research on motor control or cognitive functioning. Participants were eligible for inclusion if a modified version of the PAR-Q [20] revealed no complaints (i.e., high blood pressure, heart failure, orthopedic indications or any kind of influenza). An a priori power analysis with an actual power of 0.9 using an ANOVA design including one group, two experimental conditions with 6 repeated measurements yielded a total sample size of *N* = 18 (α = 0.05; critical *F* = 1.66). Effect size (eta^2^ = 0.82) was estimated based on a study by Simoneau and colleagues [15].

**Table 1:** Participants’ characteristics (mean ± standard deviation).

	total	male	female
	(*N* = 20)	(*n* = 10)	(*n* = 10)
Age [yrs]	23.7 ± 2.7	23.2 ± 1.9	24.2 ± 3.2
Height [cm]	176.8 ± 9.1	182.7 ± 8.1	170.8 ± 5.3
Mass [kg]	68.7 ± 11.1	75.3 ± 10.0	62.1 ± 7.9
BMI [kg/m^2^]	21.9 ± 2.5	22.6 ± 2.8	21.3 ± 2.0
SMM [%]	48.4 ± 4.8	52.1 ± 2.7	44.7 ± 3.3
Body Fat [%]	14.5 ± 7.4	9.2 ± 4.8	19.7 ± 5.7
Physical activity [h/week]*	13.2 ± 7.1	11.4 ± 6.9	14.9 ± 7.1

Note: BMI = body-mass-index; SMM = skeletal muscle mass

*Physical activity was assessed using a self-reported questionnaire including overall physical activity during a normal week, everyday physical activity, and sports activity in and outside of organized clubs [21]

### Experimental procedure

The experiment was conducted on two test days, one week apart. Prior to the first session, body height was assessed using a wall-mounted stadiometer. In addition, body mass and body composition (i.e., muscle mass, body fat) were registered by means of a bioimpedance analysis system (InBody 720, BioSpace, Korea). During the first test session, participants conducted a modified incremented all-out test on a treadmill (h/p/cosmos GmbH, Nussdorf, Germany) and performed one-legged stance experiments during breaks between treadmill stages (fatigue condition). During the second session, participants completed the same experimental protocol without being fatigued (control condition, i.e., no treadmill running). The fatigued condition was conducted prior to the control condition to get a reliable number of stages that participants endured during the treadmill test. Subsequently, the number of completed treadmill stages during the fatigued condition was used to replicate the protocol for the control session. During control, subjects were seated on a chair for 3 min instead of running the treadmill. Thus, both conditions (fatigue and control) were equal in duration and order of measurements.

### Fatigue protocol

Subjects were fatigued using a modified all-out incremented treadmill. Initial treadmill speed was set to 6 kilometers per hour (km/h) and increased by 2 km/h every 3 minutes [22]. At the end of each stage, a break of 3 minutes was provided during which lactate levels [mmol/l], heart rate [bpm], rate of perceived exertion (RPE) and the postural tasks were registered. Participants conducted the one-legged-stance during ST (i.e., one-legged-stance only) and DT conditions in counterbalanced order. Time lag between those two tasks was kept as short as possible. During the DT condition, participants performed the one-legged-stance while concurrently subtracting serial 3s, starting from a randomly selected number between 300 and 900. The fatigue protocol was terminated when subjects were subjectively exhausted, as indicated by maximum RPE. Heart rate was recorded using a wearable heart rate monitor (Polar GmbH, Germany). Twenty μL arterialized capillary blood were collected from the participants’ ear lobe and analyzed for lactate concentration. The force platform to register postural control was located next to the treadmill. Hence, there was hardly any time-lag between the fatiguing activity on the treadmill and the measurements of postural control (time from the end of the treadmill stage to the start of the postural control tasks amounted to approx. 25–30 s.).

### Assessment of static postural control

Postural control was assessed during one-legged stance on the dominant leg using a 3D force platform (AMTI, USA). Total Center-of-Pressure (CoP) displacements were calculated according to the following formula:

$CoP[mm]=\sqrt{CoP_{AP}{}^{2}+CoP_{ML}{}^{2}}$. *CoP*_*AP*_ represents CoP displacements in anterior-posterior and *DTC*_*ML*_ represent CoP displacements in medio-lateral direction. In addition, CoP velocity (in m/s), indicating the total distances covered by the CoP divided by the duration of the sampled period and sway area (in mm^2^), representing the ellipse area covered by the trajectory of the CoP with a 95% confidence interval were calculated [23]. The dominant leg was determined using the lateral preference inventory [24]. Participants were asked to stand on their dominant leg, hands akimbo and gaze fixated on a wall approximately 2.5 meters apart. Data was acquired over a time interval of 30 s at a sampling rate of 1,000 Hz. Previously, high intra- (ICC = 0.97; 95% CI: 0.91–0.99) and intersession (ICC = 0.94; 95%CI: 0.84–0.98) reliability were reported for the one-legged stance [25].

### Assessment of ventilation during postural control

**Fig 1:**
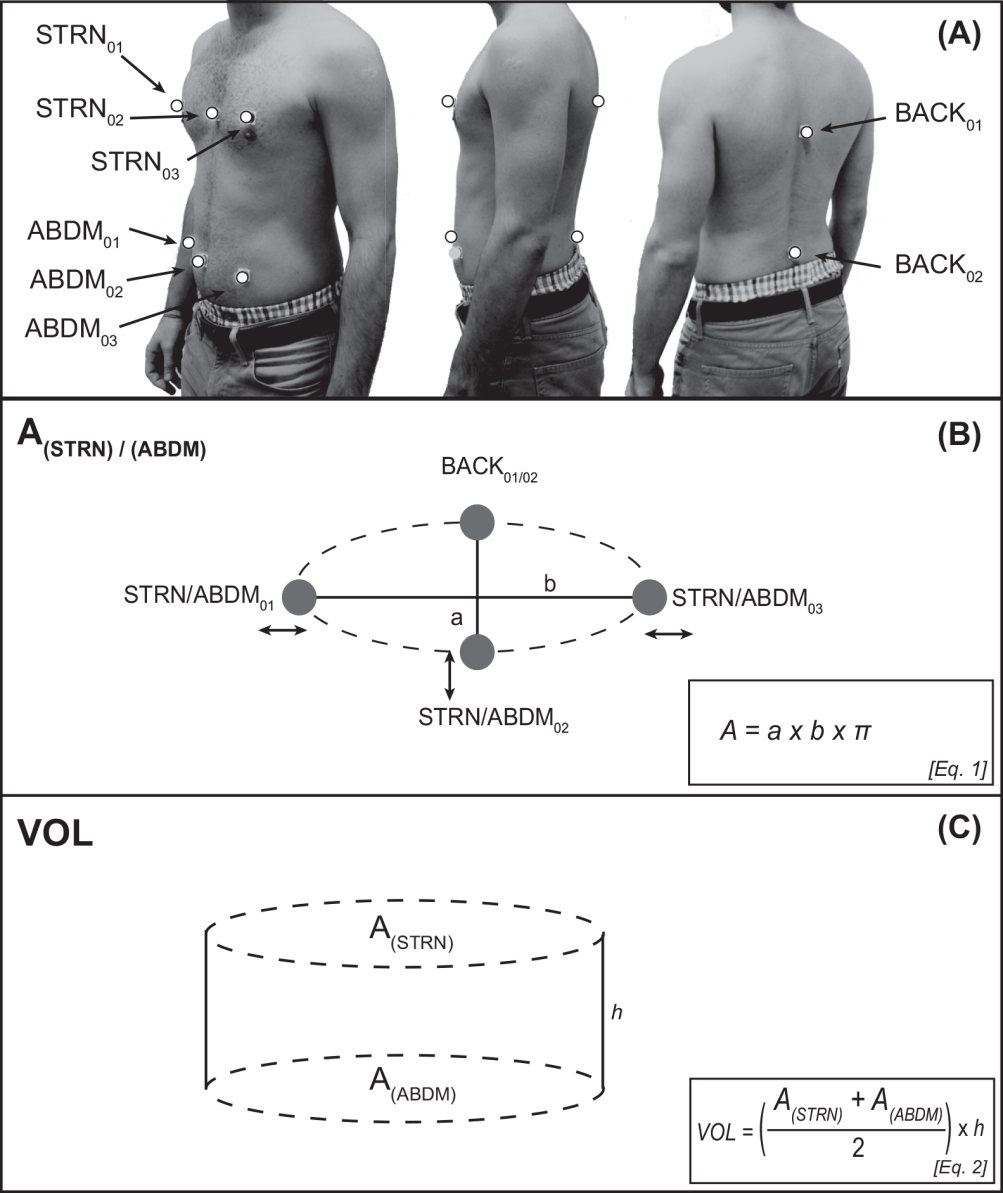
Marker setup and theoretical approach to calculate subjects’ ventilation. Fig 1A shows marker placements (white dots) used in the experiments in frontal, lateral and rear view. Fig 1B displays the theoretical approach used to calculate subjects’ chest volume. Surface areas of the thoracic and abdominal portion were calculated separately according to Eq. 1, and Fig 1C shows the calculation of a cylinder using Eq. 2 that closely matched subjects’ chest volume.

The treadmill-based fatigue protocol produced increased breathing-movements. These pronounced breathing-movements are known to affect postural control by increasing body sway [18, 19]. In order to disentangle breathing-related effects from fatigue-related effects, participants’ abdomino-thoracic ventilation was assessed. This was realized using an 8-camera motion capture system (Vicon Motion Systems, UK) and 8 reflective markers to determine ventilation at a sampling frequency of 100 Hz. Trajectories were filtered with a second order low-pass Butterworth filter using a cut-off frequency of 10 Hz. Marker positions are displayed in Fig 1A. We used the marker positions to calculate upper body (chest) volume in order to yield a proxy of ventilation. Human respiration is a combination of thoracic and abdominal breathing, thus we calculated the surface (A) of an ellipse around the sternum (i.e., thoracic proportion; markers: STRN_01_, STRN_02_, STRN_03_, and BACK_01_) and abdomen (abdominal portion; marker: ABDM_01_, ABDM_02_, ABDM_03_, and BACK_02_) using the formula shown in Equation 1 (Fig 1B), where “*a*” is the half-axis of the ellipse in AP direction, “*b*” is the half-axis in ML direction. Subsequently, we calculated the volume of a cylinder to approximate upper body volume (VOL) using the formula displayed in Equation 2 (Fig 1C), where “*A*_*(STRN)*_”is the surface of the thoracic ellipse, “*A*_*(ABDM)*_”the surface of the abdominal ellipse, and “*h*” the difference between STRN_02_ and ABDM_02_. We defined ventilation as the breathing-related change in chest volume over the course of the 30 s one-legged stance periods.

### Assessment of cognitive performance

The cognitive interference task used during DT comprised serial subtractions of 3s, starting from a randomly selected number between 300 and 900. Subjects were instructed to perform the cognitive and motor tasks as thorough as possible (i.e., no task prioritization). In addition, the interference task was performed in ST condition at the beginning of each experimental session (i.e., once during the fatigue condition and once during the control condition). Subjects were seated in a chair and asked to perform as many calculations as possible during 30 s. If a subject miscalculated, the false calculation was noted. When correctly continuing the serial subtractions, only one error was documented (i.e., no consequential errors were registered). The number of accurate calculations was used for further analysis.

Sustained attention was assessed before and after the balance protocol using a computerized version of the d2 test [26]. Sequences of letters (d and p), each enclosed by 0, 1 or 2 commas above and/or 0, 1 or 2 commas below (e.g.: d”) were presented. The letter that subjects momentarily processed was framed by a rectangle that switched to the next letter immediately after the subjects’ response. Subjects were asked to press a button with their left index finger when the letter d surrounded by two commas was highlighted, and to press a button with their right index when any other letter-comma-combination (e.g., p’) was highlighted. In total, 12 trials of 30 s each were displayed. Sustained attention was quantified as number of correctly marked target letters minus number of incorrectly marked target letters. The d2-attention-test shows a high test-retest reliability (ICC = 0.84; 95% CI: 0.74–0.89) [26].

### Statistical analyses

Data are presented as means and standard deviations. Participants finished the fatigue protocol at different stages according to their physical capacities. Thus, data are presented as percentage of maximum performance (i.e., 0%, 20%, 40%, 60%, 80%, and 100%). To estimate the effects of fatigue on heart rate and lactate levels, we performed separate 2 (Condition: fatigue vs. control) x 6 (Stage: 0–100%) analyses of variance (ANOVA) and a 2 (Condition: fatigue vs. control) x 2 (Attention: ST vs. DT) x 6 (Stage: 0–100%) analyses of variance (ANOVA) for ventilation. Subsequently, a factor analysis was calculated using unrotated factor solution for the ventilation data (from each stage and each ST and DT condition). We thus reduced the dimensions of ventilation data to yield one single factor. This factor was included as a covariate in the analyses of CoP measures [27]. To analyze ventilation-adjusted effects of fatigue and additional attentional demand on postural control, we performed 2 (Condition: fatigue vs. control) x 2 (Attention: ST vs. DT) x 6 (Stage: 0–100%) analyses of co-variance (ANCOVA). Performance of the cognitive interference task across conditions and stages was analyzed using a 2 (Condition: fatigue vs. control) x 7 (Stage: 0–100%, plus one seated trial) ANOVA and cognitive performance was analyzed using a 2 (Condition: fatigue vs. control) x 2 (Time: pre/post) ANOVA. Post-hoc tests included the Bonferroni-adjusted α and were conducted to identify comparisons that were statistically significant. All data were tested for normal distribution using one sample Kolmogorov-Smirnov tests. Effect sizes were determined by calculating Cohen’s *d*. Cohen’s d is a measure that defines whether a difference is of practical concern. Cohen’s *d* values are classified as followed: 0.00 ≤ *d* ≤ 0.49 indicate small, 0.50 ≤ *d* ≤ 0.79 indicate medium, and *d* ≥ 0.8 indicate large effects [28]. All analyses were calculated using Statistical Package for Social Sciences (SPSS) version 22.0 (IBM Corp., New York, USA) and significance levels were set at α = 5%.

## Results

Physiological data (lactate, heart rate, RPE), CoP data and ventilation data were normally distributed in each of the registered conditions (all p > 0.35).

### Physiological measures (blood lactate concentration, heart rate)

**Fig 2:**
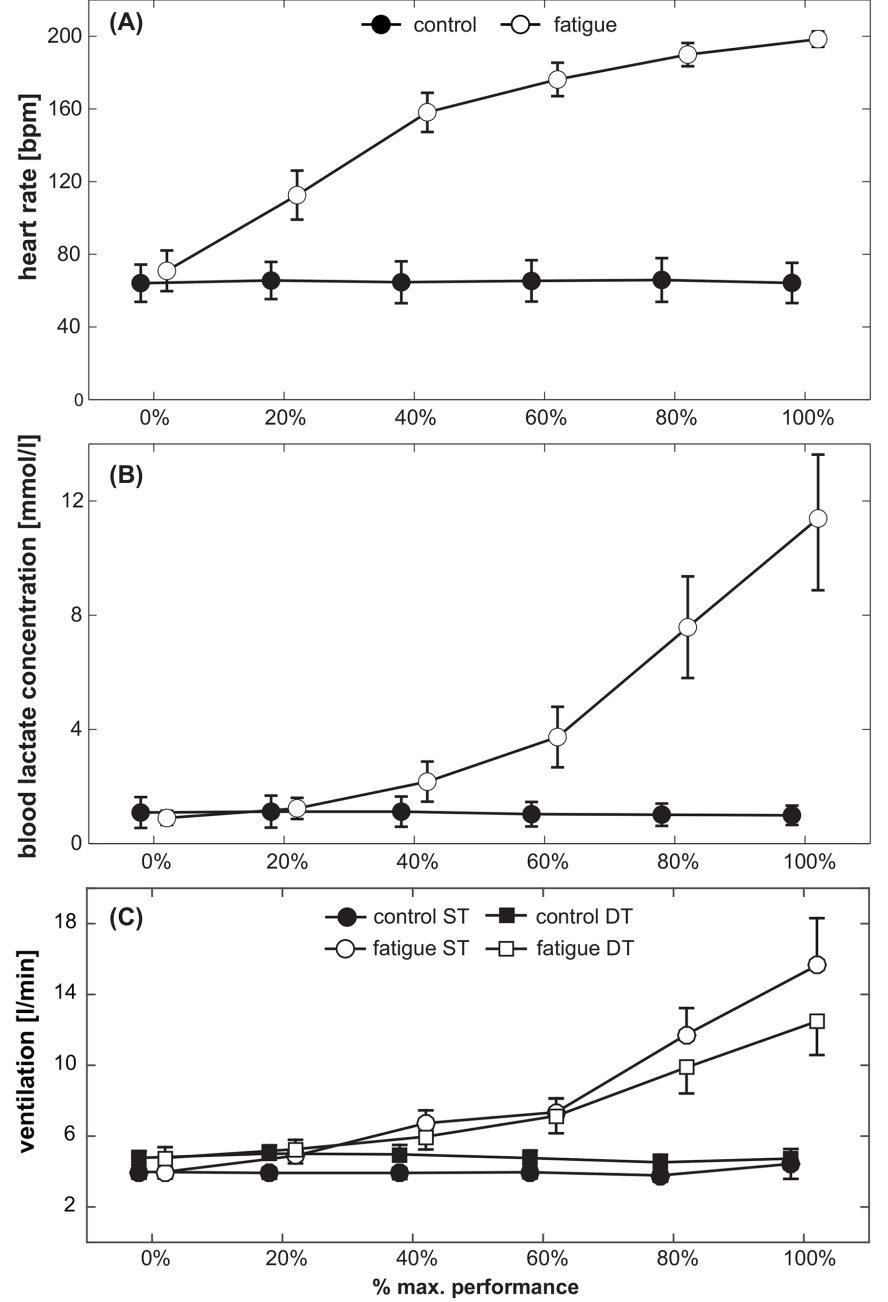
Subjects’ (A) heart rate, (B) blood lactate levels and (C) ventilation during each of the 6 different stages, displayed separately for the fatigue condition (white symbols) and the control condition (black symbols). For ventilation, data is separately displayed for ST (circles) and DT (squares) conditions. Symbols represent mean values; error bars the respective 95% confidence interval.

Subjects finished the all-out protocol between stages 5 and 10 which corresponds to maximum running speeds of 14 to 24 km/h. Heart rates and blood lactate levels are displayed in Fig 2A and 2B. ANOVA for heart rate yielded significant main effects of Condition (F_(1,19)_ = 2040.2, *p* < 0.001, *d* = 21.0) and Stage (F_(5,95)_ = 492.4, *p* < 0.001, *d* = 10.2). Heart rate significantly increased during the fatigue compared to the control condition and with each completed stage. Also, a significant Condition x Stage interaction was observed (F_(5,95)_ = 534.5, *p* < 0.001, *d* = 10.6).

Similar results were obtained for blood lactate levels. The analysis revealed significant main effects of Condition (F_(1,19)_ = 300.5, *p* < 0.001, *d* = 8.0) and Stage (F_(5,95)_ = 185.8, *p* < 0.001, *d* = 6.2). This stage-related increase was more pronounced during the fatigue compared to the control condition, i.e., significant Condition x Stage interaction (F_(5,95)_ = 188.5, *p* < 0.001, *d* = 6.3). Post-hoc test indicate that heart rate (all p < 0.001) and lactate concentration increased with every stage (all p < 0.01) during the fatigue condition.

### Ventilation

Ventilation is displayed in Fig 2C. ANOVA yielded significant main effects of Condition (F_(1,19)_ = 161.8, *p* < 0.001, *d* = 5.8) and Stage (F_(5,95)_ = 56.7, *p* < 0.001, *d* = 3.5). Ventilation significantly increased during the fatigue as compared to the control condition and with each completed stage. In addition, a significant Condition x Stage interaction was found (F_(5,95)_ = 84.8, *p* < 0.001, *d* = 4.2). Ventilation significantly increased with each stage during the fatigue condition but not during control (post-hoc: all p < 0.01). The main effect of Attention was not significant (F_(1,19)_ = 0.0, *p* = 0.98, *d* = 0.1), but there were two highly significant interactions involving this manipulation: Condition x Attention (F_(1,19)_ = 28.4, *p* < 0.001, *d* = 2.4) and Condition x Stage x Attention (F_(5,95)_ = 4.6, *p* < 0.001, *d* = 1.0), indicating that the stage-related increase in ventilation was more pronounced during ST compared to DT.

### Static postural control

**Fig 3:**
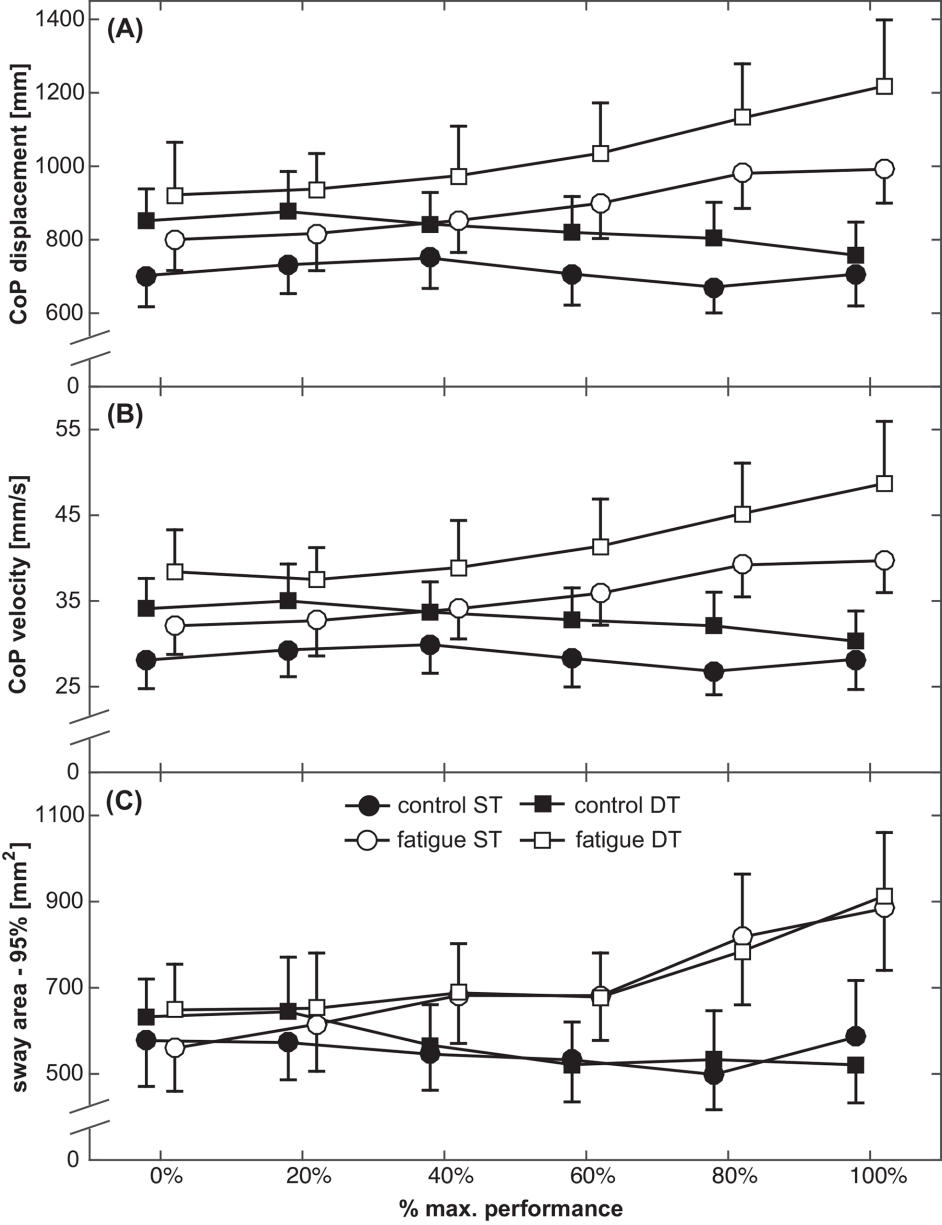
Subject’s (A) total CoP displacements, (B) mean CoP velocity and (C) 95% sway area, displayed separately for the control condition (black symbols) and the fatigue condition (white symbols) during ST (circles) and DT (squares) conditions. Symbols represent means; error bars indicate the respective 95% confidence interval.

Total CoP displacements, CoP velocity and sway area are displayed in Fig 3A–3C. ANOVA yielded significant main effects of Condition (F_(1,18)_ = 78.7, *p* < 0.001, *d* = 4.2) and Stage (F_(5,90)_ = 7.2, *p* < 0.001, *d* = 1.3) for ventilation-adjusted CoP displacements, indicating higher displacements during the fatigue compared to the control condition. A significant Condition x Stage interaction (F_(5,90)_ = 16.9, *p* < 0.001, *d* = 1.9) showed that the increase was more pronounced in the fatigue condition. Post-hoc tests indicated that CoP displacements at 80% (*p* = 0.01, *d* = 2.1) and at 100% of subjects’ maximal performance (*p* = 0.004, *d* = 2.5) significantly differed from the first (non-fatigued) stage. Further, the analysis revealed a significant main effect of Attention (F_(1,18)_ = 17.1, *p* = 0.001, *d* = 1.9). Total CoP displacements were higher in DT compared to ST. No significant Condition x Attention (F_(1,18)_ = 2.1, *p* = 0.16, *d* = 0.7), Attention x Stage (F_(5,90)_ = 1.3, *p* = 0.97, *d* = 0.2), and Condition x Attention x Stage (F_(5,90)_ = 1.8, *p* = 0.12, *d* = 0.7) interactions were found.

Similar results were found for CoP velocity. ANOVA yielded significant main effects of Condition (F_(1,18)_ = 74.5, p < 0.001, d = 4.1), Stage (F_(5,90)_ = 5.7, p < 0.01, d = 2.8) and a Condition x Stage interaction (F_(5,90)_ = 7.5, p < 0.001, d = 3.3). Post-hoc tests indicated that CoP velocity at 80% (p = 0.03, d = 2.7) and at 100% of subjects’ maximal performance (p = 0.008, d = 2.5) significantly differed from the non-fatigued stage. The main effect of Attention (F_(1,18)_ = 17.3, p = 0.001, d = 2.0) indicated that CoP velocity was higher in DT compared to ST condition. No significant Condition x Attention (F_(1,18)_ = 3.1, p = 0.09, d = 0.8), Attention x Stage (F_(5,90)_ = 0.3, p = 0.92, d = 0.6), and Condition x Attention x Stage (F_(5,90)_ = 1.5, p = 0.25, d = 1.5) interactions were found.

For sway area, a significant main effect of Condition (F_(1,18)_ = 49.6, p < 0.001, d = 3.3), and a Condition x Stage interaction (F_(1,18)_ = 4.6, p = 0.01, d = 2.6) were found, indicating that sway area increased with each stage during the fatigue condition. Post-hoc tests indicated that sway area at 100% of subjects’ maximal performance (p = 0.03, d = 1.9) significantly differed from the non-fatigued stage. No significant Stage (F_(1,18)_ = 2.3, p = 0.09, d = 1.8), Attention (F_(1,18)_ = 0.6, p = 0.46, d = 0.4), Condition x Attention (F_(1,18)_ = 0.01, p = 0.94, d = 0.0), Attention x Stage (F_(5,90)_ = 0.7, p = 0.67, d = 1.0), and Condition x Attention x Stage (F_(5,90)_ = 0.3, p = 0.91, d = 0.6) interactions were found.

### Cognitive performance

**Fig 4:**
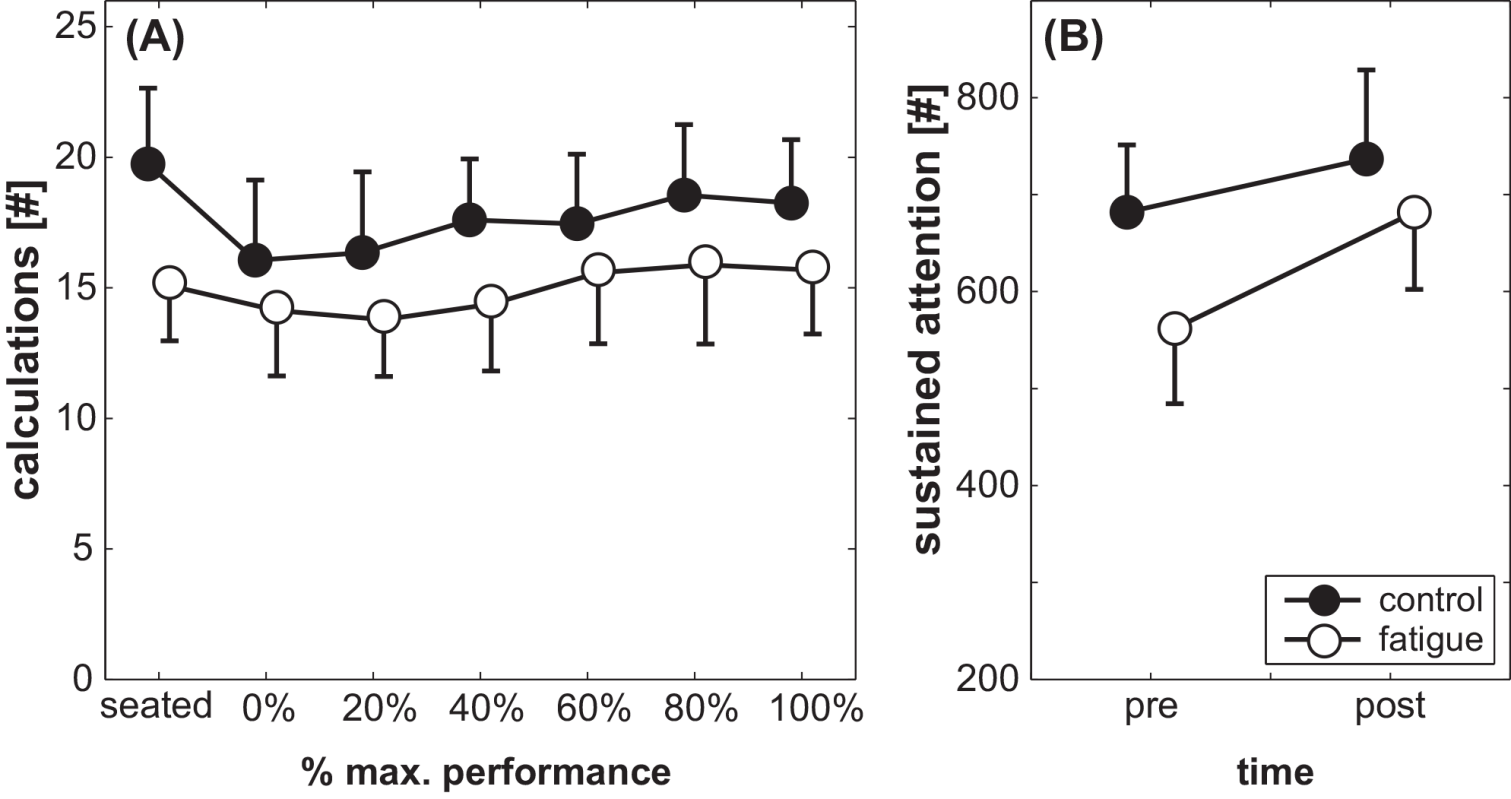
Subject’s performance in (A) the cognitive task at each stage and (B) their d2-attention-test score before and after the fatigue and control condition, displayed separately for the control condition (black symbols) and the fatigue condition (white symbols). Symbols represent means; error bars indicate the respective 95% confidence interval.

Performance in the cognitive interference task (Fig 4A) showed a significant main effect of Condition (F_(1,19)_ = 29.2, *p* < 0.001, *d* = 2.5). During the control condition, subjects completed more correct calculations as compared to the fatigue condition. Also, performance improved with advanced protocol duration, i.e., significant main effect of Stage (F_(6,114)_ = 7.6, *p* < 0.001, *d* = 1.3). However, no significant Fatigue x Stage interaction was found (F_(6,114)_ = 1.8, *p* = 0.10, *d* = 0.6).

Sustained attention (Fig 4B) showed a significant main effect of Condition (F_(1,40)_ = 13.5, *p* < 0.01, *d* = 1.2) and Time (F_(1,40)_ = 187.5, *p* < 0.001, *d* = 4.3). Attention was higher in the control condition compared to the fatigue condition and improved after the balance protocol compared to baseline. Additionally, a significant Condition x Time interaction was observed (F_(1,40)_ = 25.6, *p* < 0.001, *d* = 1.6). The increase in cognitive performance was more pronounced in the fatigue condition compared to the control condition (cf. Fig 4B).

## Discussion

To our knowledge, this is the first study that investigated the effects of gradually increasing levels of fatigue on ST and DT postural control in young adults. We additionally incorporated physiological (lactate, heart rate) and cognitive (d2-attention-test) measures to explain our findings on a peripheral and central level, respectively. Pronounced breathing-movements have been shown to affect postural control [18, 19]. Our study is one of the first studies that unraveled breathing-related effects from fatigue-related effects on postural control by controlling for ventilation. Results showed that (1) postural control was impaired following the fatigue protocol; (2) postural control was compromised during DT compared to ST; (3) impairments in ST and DT postural control increased with advancing levels of fatigue; and (4) fatigue did not further impede postural control during DT compared to ST. As expected, heart rate, lactate levels and ventilation significantly increased with fatigue, showing that the modified stage protocol used was suitable to induce fatigue.

### Effects of fatigue on postural control

Total CoP displacements, velocity and sway area increased with increasing levels of physical exhaustion. This finding is in line with previous research showing impaired balance performance in healthy young adults following local [6, 7, 29] and whole-body fatigue [7, 30]. In our study, postural control deteriorated by 23–41% at maximal exertion. Previous studies found similar fatigue-related deteriorations. Following a whole-body treadmill protocol, sway velocity of young adults during one-legged stance increased 14–38% [7]. Additionally, we were able to show that postural control was significantly deteriorated when subjects reached at least 80% of their maximal performance compared to the non-fatigued condition. This indicates that decrements in postural control emerge at rather high levels of physical fatigue. Fatigue-related decrements in postural control may be explained by peripheral changes at the level of the muscle (i.e., the neuromuscular junction, the sarcolemma, accumulation of metabolites and depletion of substrates) and by insufficient drive of the central nervous system to the motor neurons [10]. Fatigue induces lower firing rates of motor units [31, 32] and a reduction in the number of active motor neurons [33]. On the afferent side, diminished synaptic (i.e., decreased H-reflex amplitude) [11] and proprioceptive (i.e., exaggerated force generation in force matching tasks) [12] feedback has been shown. In addition, position sense in the ankle is impaired when subjects are fatigued [34], which impairs postural control. Lactate levels are markers of peripheral fatigue following exercise [35] and indicate that the metabolism is not able to eliminate lactate adequately, i.e., the muscles fatigue. Lactate levels in our study significantly increased at each stage during the fatigue protocol, indicating that we were able to fatigue the peripheral (i.e., muscular) system of our subjects. These alterations ultimately lead to performance decrements in postural control and decreased efficiency of the muscle [36]. In other words, the neuromuscular system is less capable of generating and controlling adequate muscular output.

### Effects of attentional demand on postural control

Our findings on the effects of attentional demand on postural control are predominantly in line with previous research (i.e., two out of three parameters showed attention-related performance decrements). Many studies showed that postural sway and sway velocity increased when performing attention-demanding interference tasks during standing [2, 4]. Increased postural sway and a reduction in the speed and accuracy of responses in the interference task were found in young adults while simultaneously performing a serial subtraction task [2, 5]. Additionally, sway frequency (i.e., speed of body sway) increased while concurrently counting backwards [4]. When two tasks are concurrently performed with one task demanding postural control and the other task requiring cognitive processing, a decrement in performance of one or both tasks can be observed. This deficit is most likely caused by limited cognitive capacities (i.e., central overload) [37]. The performance of cognitive interference tasks affords attentional demand and limits attentional resources which are needed to control the postural task [38].

### Effects of fatigue on dual-task postural control

There was no reliable evidence that the gradual increase in physical fatigue further impeded postural control during DT. There are only a few studies available that examined DT balance performance after physical exhaustion. Bisson and colleagues [17] investigated the effects of ankle and hip muscle fatigue on balance performance while concurrently performing a reaction time task in young adults. Results showed increased sway velocity and reduced reaction times during DT compared to ST, but no further impairment when subjects were locally fatigued. The authors argued that localized fatigue of the ankle and hip muscles was not sufficient to impair postural control [17]. In accordance with the central overload theory [37], one might state that the attentional demand needed to control motor processes after being fatigued results in an overload of cognitive capacities. In contrast, Simoneau and colleagues examined the effects of a whole-body fatigue protocol on DT balance performance in young adults [15]. Results showed impaired balance performance (i.e., increased CoP velocity) and increased attentional demand (i.e., increased reaction times), suggesting that more cognitive resources had to be allocated to the balance task during the fatigued compared to the non-fatigued condition. However, none of the latter two studies investigated the additional effects of gradually increasing levels of fatigue on DT balance performance. Thus, our study is the first to examine the relationship between gradual levels of fatigue on ST as well as DT postural control in young adults. In our study, subjects were completely exhausted, as indicated by heart rate, lactate levels and their RPE. However, we did not observe further decrements in DT balance performance compared to non-fatigued DT performance. For instance, attentional demand increased CoP displacements by 130 ± 42 mm on average across all treadmill stages (ST: 801 ± 109 mm; DT: 931 ± 138 mm; cf. Fig 3A) during fatigued and non-fatigued condition, indicating that increasing levels of fatigue did not further impede DT postural control. One might argue that balance control is primarily influenced by physical exhaustion on a peripheral level, as indicated by increased lactate levels in our study, and to a smaller amount by additional cognitive demands (i.e. central mechanisms). The effect sizes found in our study confirm this assumption. Cohen’s *d* was 4.2 for the effect of fatigue, but only 1.9 for the effect of attentional demand during the balance task. Therefore, it is possible that a more demanding cognitive interference task, which puts more stress on central mechanisms (i.e., cognitive capacities), might induce the predicted deterioration during DT following exhaustion (cf. [39] to compare various effects of different cognitive interference tasks on balance performance and [40] to compare effects of a subtraction task during walking). Another explanation can be derived from studies showing positive effects of acute physical exercise on cognitive functions [41]. We were able to show that performance in a sustained attention task (measured by the d2-attention-test) improved following fatigue, a phenomenon that was less distinct in the control condition. We argue that cognitive functions benefit from physiological adaptations (i.e., increased cerebral blood flow, attentional arousal) after acute exercises [41]. This beneficial effect on cognitive performance mitigates the expected impairments in cognitive function following fatigue and may thus explain the absence of further fatigue-related deteriorations in DT compared to ST postural control.

### Study limitations

Our study has some limitations that need to be considered. First, our subject group was recruited from sports science students with an overall physical activity of 13.2 hours/week. Thus, our population can be deemed highly active and might only represent a specific group not including non-fit young adults or even older adults. Second, the order of experiments (i.e., fatigue and control condition) was not randomized. We always performed the fatigue condition first, which might have caused order effects in our results. The same limitation might affect the d2-attention task. Learning the task during the fatigue session improved performance. This learning effect was carried over to the control session, leaving less room for improvement during the latter condition. Lastly, the cognitive task (i.e., subtraction of serial 3s) might not have been demanding enough to further impede subjects’ postural control. Future studies should use more demanding cognitive interference tasks to confirm our second hypothesis (cf. [42, 43]).

## Conclusions

This study illustrates that attentional demand and physical fatigue affect postural control in healthy young adults in a similar manner. More specifically, fatigue applied in the form of an all-out treadmill protocol deteriorated balance performance in both, ST and DT situations. This suggests the presence of sufficient attentional resources to maintain both motor and cognitive control with increasing fatigue when performing a motor and a cognitive task simultaneously. This is of particular interest in activities when both, attention and fatigue influence motor performance (i.e., in team sports).
